# Carrier-Free Peptide–Daunorubicin–Small Interfering RNA Nanoassembly for Targeted Therapy of Acute Myeloid Leukemia

**DOI:** 10.34133/cbsystems.0436

**Published:** 2025-11-05

**Authors:** Haiyin Yang, Xi Yu, Zhitong Guo, Songxuan Shi, Jie Wang, Shuai Guo, Bo Hu, Meihong Chai, Zhuoran Wang, Stefan Barth, Kelong Fan, Huining He, Mengjie Zhang, Yuanyu Huang

**Affiliations:** ^1^ School of Life Science; School of Interdisciplinary Science; Aerospace Center Hospital; Key Laboratory of Molecular Medicine and Biotherapy; Key Laboratory of Medical Molecule Science and Pharmaceutics Engineering; Beijing Institute of Technology, Beijing 100081, P. R. China.; ^2^ Tianjin Key Laboratory on Technologies Enabling Development of Clinical Therapeutics and Diagnostics, School of Pharmacy, Tianjin Medical University, Tianjin 300070, P. R. China.; ^3^ Xi’an Hospital of Traditional Chinese Medicine, Xi’an 710021, P. R. China.; ^4^ CAS Engineering Laboratory for Nanozyme, Key Laboratory of Protein and Peptide Pharmaceutical, Institute of Biophysics, Chinese Academy of Sciences, Beijing 100101, P. R. China.; ^5^ South African Research Chair in Cancer Biotechnology, Institute of Infectious Disease and Molecular Medicine (IDM), Department of Integrative Biomedical Sciences, Faculty of Health Sciences, University of Cape Town, Cape Town 7925, South Africa.; ^6^ School of Medical Engineering; School of Interdisciplinary Science; Affiliated Zhuhai People’s Hospital; Beijing Institute of Technology, Zhuhai 519088, P. R. China.; ^7^ Advanced Technology Research Institute, Beijing Institute of Technology, Ji’nan 250100, P. R. China.

## Abstract

Acute myeloid leukemia (AML) continues to represent a substantial unmet therapeutic need in clinical practice. In recent years, peptide–drug conjugates and small interfering RNA (siRNA) drugs have gained considerable attention due to their impressive clinical progress in treating various diseases. In this study, we designed a carrier-free “3-in-1” peptide–daunorubicin–siRNA (PDR) nanoassembly, which combines a cell-penetrating and tumor-suppressing peptide, a daunorubicin (DNR) prodrug, and siRNA targeting the LILRB4 gene. After optimizing the molar ratio among peptide, DNR prodrug, and siRNA, we identified the most potent PDR formulation, which exhibited excellent intracellular uptake efficiency, primarily through caveolin-mediated endocytosis, in THP-1 cells. The pH-responsive bond in the DNR prodrug facilitated the endosomal escape of siRNA, leading to significant gene repression of LILRB4. Additionally, the tumor-suppressing peptide p16^MIS^ effectively inhibited the transition of cells from the S phase to the G2/M phase and induced apoptosis. In a leukemia mouse model, PDR efficiently suppressed leukemia cell invasion, prolonged survival, and reduced leukemia cell infiltration in the bone marrow. Notably, silencing LILRB4 not only promoted T cell maturation in spleen and lymph nodes but also enhanced T cell infiltration in tumor tissues. This study offered a highly promising therapeutic strategy for AML and other diseases.

## Introduction

Acute myeloid leukemia (AML) is a group of malignant disorders originating from the clonal proliferation of myeloid hematopoietic progenitor or stem cells [1]. It primarily affects adults, exhibits a relatively high incidence, and is relating to a poor prognosis, with 5-year survival rate of only 25% to 40%. Although the current “7 + 3” induction chemotherapy regimen, consisting of an anthracycline combined with cytarabine, remains the standard treatment for AML [2,3], its nonselective cytotoxic mechanism often leads to severe myelosuppression and organ toxicity, thereby limiting long-term therapeutic efficacy [4].

AML demonstrates considerable biological heterogeneity and, based on the latest World Health Organization (WHO) classification, is divided into 11 distinct subtypes [5]. The complex pathogenesis of AML contributes to its overall poor prognosis [6]. Multiple driver gene mutations, including FLT3-ITD, CEBPA, IDH1/2, and DNMT3A, have been identified as being closely associated with AML progression [7–10]. Targeted therapies developed based on these mutations have shown promising therapeutic potential. However, approximately 30% to 40% of AML patients still lack clearly defined druggable targets—particularly those with monocytic subtypes such as M4 and M5, which are characterized by high aggressiveness and poor prognosis. Therefore, developing precision therapeutic strategies that involve multi-mechanistic synergies represents a key research direction for advancing the treatment of AML.

LILRB4 has recently been defined as a novel immune checkpoint, and its potential relevance in AML has increasingly attracted attention [11–13]. LILRB4 has been shown to drive immune evasion and relapse in AML by inhibiting T cell activity and promoting tissue infiltration via the ApoE–LILRB4–SHP2–NFκB (nuclear factor κB) pathway. It is positively expressed in over 80% of M4/M5 cases and is closely linked to poor overall survival [14,15]. The monoclonal antibody IO-202 targeting LILRB4 has entered phase I clinical trials (NCT04372433), and LILRB4-based therapies such as CAR-T (chimeric antigen receptor T) cells and antibody–drug conjugates have also demonstrated promising potential [12,16], further supporting its role as a specific therapeutic target.

Small interfering RNA (siRNA) technology enables the silencing of virtually any gene through sequence-specific base pairing, thereby greatly expanding the range of druggable targets [17]. Although several siRNA-based drugs have been approved [18–24]—primarily for the therapy of rare diseases—their therapeutic potential in oncology is equally promising [25,26]. Despite its therapeutic potential, siRNA still faces several major hurdles: Its structure is inherently unstable and prone to nuclease degradation; it is rapidly cleared from the bloodstream [27,28]; its strong negative charge hinders efficient transmembrane transport; and most internalized siRNA becomes trapped in endosomes, resulting in extremely low cytoplasmic release efficiency [29–31]. Therefore, developing nucleic acid delivery systems capable of specific targeting, efficient cellular uptake, and effective endosomal escape holds great promise [28,32].

**Fig. 1.**
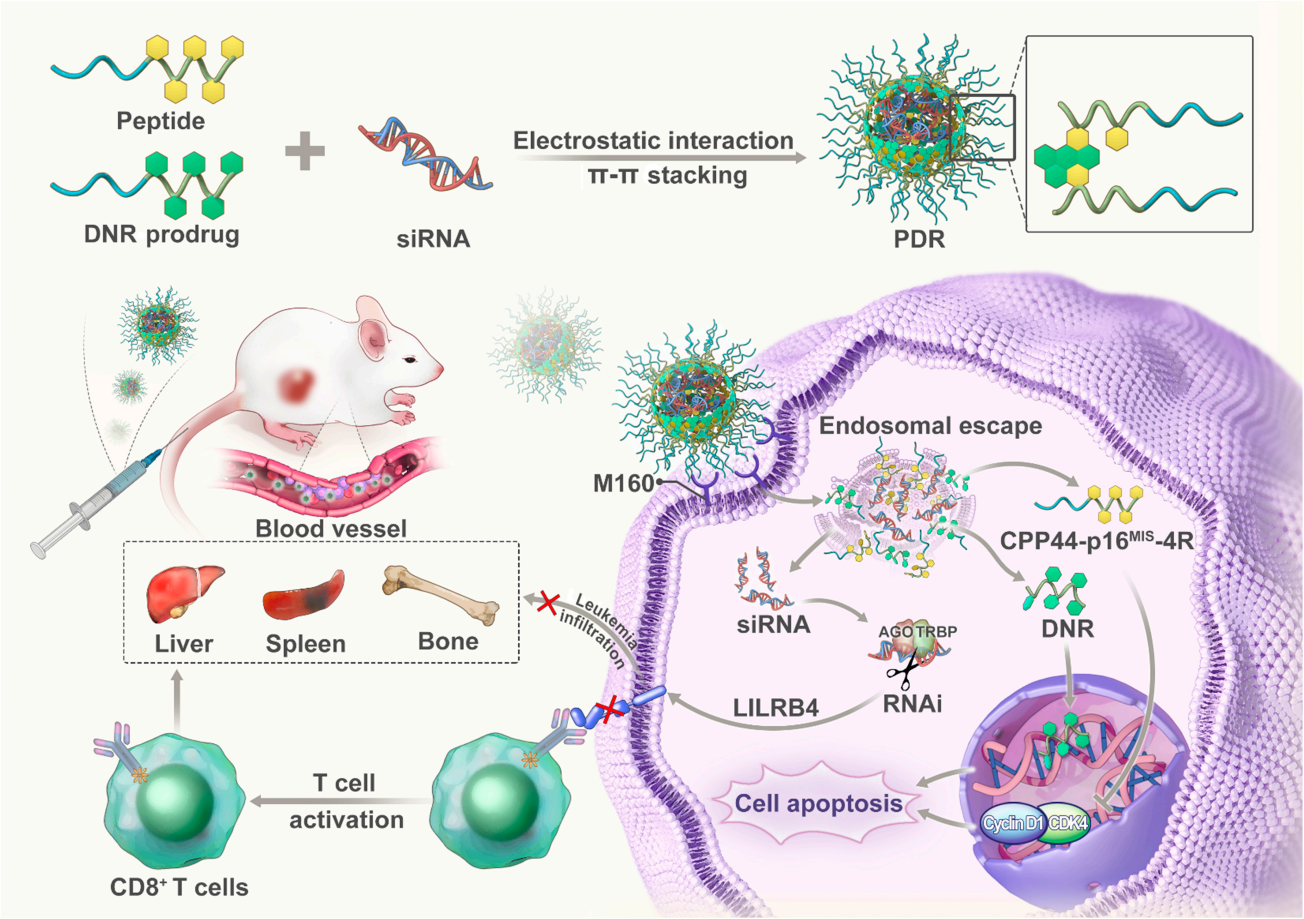
Schematic illustration of PDR mediated synergetic leukemia suppression.

To this end, we designed and constructed a carrier-free tumor-suppressing peptide–daunorubicin–siRNA (PDR) nanoassembly (Fig. 1 and Fig. S1). PDR is composed of 3 functional components: (a) a multifunctional tumor-suppressing peptide (CPP44-p16^MIS^-FFY-4R), in which CPP44 is a novel AML-targeting cell-penetrating peptide that enables efficient endocytosis and selective delivery [33]; p16^MIS^ mimics the critical functional domain of the p16 protein and inhibits cyclin-dependent kinase 4/6 (CDK4/6) activity to induce G1-phase cell cycle arrest [33]; FFY (phenylalanine-phenylalanine-tyrosine) is an aromatic self-assembling tripeptide that drives the core assembly of the daunorubicin (DNR) prodrug via π–π stacking; and the 4 arginine residues (4R) bind siRNA through electrostatic interactions and facilitate endosomal escape; (b) a pH-sensitive DNR prodrug (PEG-Hyd-DNR), in which the hydrazone bond degrades in the acidic endosomal environment to enable drug release and endosomal escape; and (c) siLILRB4, an siRNA that silences the LILRB4 gene to relieve immunosuppression and enhance anti-leukemic immune responses. The 3 components spontaneously self-assemble into PDR nanoparticles in aqueous solution through electrostatic interactions and hydrophobic/π–π stacking forces. It has been reported that the cellular uptake of CPP44 in AML is mainly facilitated by M160 receptor-dependent endocytosis [33]. After internalization, PDR enables stimulus-responsive endosomal escape and exert coordinated anti-leukemic therapeutic effects. Compared with current mainstream siRNA delivery systems, such as lipid nanoparticles (LNPs) [34,35], polymer-based carriers [36], and GalNAc conjugation technology [37], the PDR system demonstrates significant advantages in achieving precise delivery to the target site. Conventional systems like LNPs and polymeric carriers often rely on nontherapeutic components as delivery scaffolds. These empty carriers lack pharmacological activity and may trigger unwanted immune or inflammatory responses in vivo, thereby increasing formulation complexity and the risk of potential side effects. In contrast, the PDR system consists of 3 functional components, each possessing therapeutic properties. They not only work synergistically to enhance targeting and stability during delivery but also exert combined therapeutic effects through multiple mechanisms. As a result, PDR improves delivery efficiency while enhancing overall treatment efficacy, offering a more efficient and safer novel strategy for siRNA therapy.

## Materials and Methods

### Reagents and materials

RPMI 1640 medium, fetal bovine serum (FBS), penicillin–streptomycin, Lipofectamine 2000, and Opti-MEM (minimum essential medium) were purchased from Thermo Fisher Scientific, USA. Polyethylene glycol 2000 (PEG-2000) and DNR were purchased from Macklin Biochemical Technology Co. Ltd. (Shanghai, China). Hoechst 33342 and LysoTracker Green DND-26 were purchased from Sigma-Aldrich. GelstainRed Nucleic acid dyes were purchased from Youyilandi Biotechnology Co. Ltd. (Suzhou, China). Micro BCA Protein Assay kit was purchased from Beyotime Biotechnology Co. Ltd. (Shanghai, China). Agarose was purchased from GEN TECH (Hong Kong, China). The LILRB4 (ILT3) antibody (ab229747, 1:5,000 dilution) was purchased from Abcam. glyceraldehyde-3-phosphate dehydrogenase (GAPDH) antibody (60004-1-Ig, 1:50,000 dilution) and β-actin (20536-1-AP, 1:10,000 dilution) were purchased from Proteintech. Anti-human phycoerythrin (PE)–CD85k (ILT3) antibody, anti-mouse phycoerythrin (PE)-CD3 antibody, anti-mouse fluorescein isothiocyanate (FITC)–CD4 antibody, and anti-mouse pacific blue (PB)-CD8a antibody were purchased from BioLegend. Hieff qPCR SYBR Green Master Mix and Annexin V-FITC/PI Apoptosis detection kit were purchased from Yeasen Biotechnology Co. Ltd. (Shanghai, China). d-Luciferin was bought from Promega Co. Ltd. (Madison, USA). All siRNA used in this study, including Cy5-siRNA (SS: 5′- UUCUCCGAACGUGUCACGUTT-3′, AS: 5′-ACGUGACACGUUCGGAGAATT-3′), siNC (SS: 5′-UUCUCCGAACGUGUCACGUTT-3′, AS: 5′-ACGUGACACGUUCGGAGAATT-3′), and siLILRB4 (SS: 5′-GAGGACAGACAGAUGGACA-3′, AS: 5′- UGUCCAUCUGUCUGUCCUCUU-3′), were provided by Suzhou Ribo Life Science Co. Ltd. (Jiangsu, China) and Suzhou Olipharma Co. Ltd. (Jiangsu, China). Polypeptide (CPP44-p16^MIS^-FFY-4R) was obtained from Xi’an Ruixi Biological Technology Co. Ltd. (Xi’an, China). The amino acid sequence was KRPTMRFRYTWNPMK-GPG-rhlvvltdlf-GP-FFYFFY-RRRR.

### Cell culture

THP-1 cells were obtained from the American Type Culture Collection (ATCC) and cultured in RPMI 1640 medium. The mouse leukemia cells with high expression of human LILRB4 gene (C1498-Luc-hLILRB4) were constructed by Misen Cell Technology Co. Ltd. and cultured in Dulbecco’s modified Eagle’s medium (DMEM). The culture medium was supplemented with 10% FBS, 2 mM l-glutamine, 100 U/ml penicillin, and 100 μg/ml streptomycin. Cells were maintained at 37 °C in a humidified atmosphere with 5% CO₂.

### Synthesis of DNR prodrug

A pH-sensitive DNR prodrug was prepared by linking DNR to PEG-2000 via a hydrazone bond, following a previously reported method. At 0 °C, PEG-2000 and triethylamine were dissolved in anhydrous dichloromethane, followed by the slow addition of *p*-nitrophenyl chloroformate in dichloromethane. The reaction was continued at 0 °C for 1 h, followed by stirring at room temperature under a nitrogen atmosphere for an additional 24 h. The resulting solution was diluted with dichloromethane and washed 3 times with brine solution. The organic layer was separated, dried over anhydrous sodium sulfate, and precipitated with ether to yield PEG-NPC (the reaction product between PEG2000 and p-nitrophenyl chloroformate). PEG-NPC (213 mg, 0.093 mmol) was then dissolved in 20 ml of dichloromethane and reacted with hydrazine monohydrate (0.93 mmol) at ambient temperature for 24 h. After washing with brine, the organic phase was collected, the solvent was removed, and the residue was dried under vacuum to afford PEG-Hyd (the reaction product between PEG-NPC and hydrazine monohydrate). Subsequently, PEG-Hyd (60 mg, 0.027 mmol) and DNR (15 mg, 0.027 mmol) were codissolved in 25 ml of anhydrous methanol and stirred overnight at 60 °C with the addition of a drop of trifluoroacetic acid as a catalyst. Following solvent removal under vacuum, the resulting material was dissolved in water and dialyzed for 24 h at 4 °C using a 1,000-Da molecular weight cutoff (MWCO) dialysis membrane. The final product was obtained by freeze-drying.

### Preparation and characterizations of PDR

PDR nanoparticles were prepared by nanoprecipitation using DNR prodrug, functionalized peptide, and siRNA in a specific ratio. Specifically, an aqueous solution of siRNA (100 μl, 40 μg) was slowly added to a mixture (300 μl) of DNR prodrug and functionalized peptide under stirring at room temperature for 15 min, with a molar ratio of DNR prodrug to functionalized peptide to siRNA of 50:10:1. The resulting mixture was dialyzed against phosphate-buffered saline (PBS) (pH 7.4) for 2 h using a 100-kDa MWCO membrane to obtain the PDR nanoparticles. Subsequently, the zeta potential, PDI, and diameter were characterized via dynamic light scattering (DLS).

To assess siRNA encapsulation, PDR nanoparticles (20 μl, containing 200 ng siRNA) were subjected to electrophoresis at 120 V for 30 min in tris–acetate–EDTA (TAE) buffer. siRNA bands were visualized using GelStain. Ultraviolet–visible (UV–vis) absorption and fluorescence spectra of free DNR, the DNR prodrug, and PDR were recorded using a Cytation 5 multimode reader (BioTek, USA).

For transmission electron microscope (TEM) analysis, 10 μl of PDR solution was added to copper grids and stirred for 30 min, washed with water, and then stained with 2% uranyl acetate for 5 min. Finally, electron microscopic images were taken using Tecnai Spirit TEM (FEI, USA) under 80 kV.

### mRNA and protein analysis of LILRB4

Reverse transcription quantitative polymerase chain reaction (RT-qPCR) and Western blot were performed to assess gene silencing efficiency of LILRB4. THP-1 cells (5 × 10^5^ or 1 × 10^6^ per well) were seeded in 12- or 6-well plates. Subsequently, cells were transfected with PDR or Lipo2000 (commercial transfection reagent, positive control) containing 100 nM siRNA for 24 h. Total RNA or protein was isolated and used for qPCR and Western blot, respectively.

### Internalization of PDR

To evaluate internalization of PDR nanoparticles in THP-1 cells, 5 × 10^5^ cells/well were seeded into 12-well plates and incubated with PDR nanoparticles (NPs) containing 100 nM Cy5-labeled siRNA in Opti-MEM for 4 h. After incubation, cells were harvested by centrifugation (300g), washed 3 times with PBS, resuspended in 400 μl of PBS, and analyzed by flow cytometry (BD Biosciences, USA). Mean fluorescence intensity (MFI) was quantified using FlowJo 10.8.1.

To investigate the uptake pathways, cells were treated with the following regents: amiloride (100 μM), genistein (1 mM), chlorpromazine (30 μM), and methyl-β-cyclodextrin (50 μM) to inhibit endocytosis. After incubation for 30 min, PDR was then added and incubated with cells for 4 h. Cells were analyzed by fluorescence-activated cell sorting (FACS) or stained with nuclei and endosomes/lysosomes for confocal laser scanning microscopy (CLSM) observation. Cy5 fluorescence intensity and colocalization with endosomal/lysosomal signals were analyzed accordingly.

### Cell viability and apoptosis assay

THP-1 cells were coincubated with PDR containing 100 nM siLILRB4 or siNC. Untreated cells were used as the Mock group. The wells containing only medium without cells were used as the Blank group. Each well received 10 μl of Cell Counting Kit-8 (CCK-8) reagent, followed by a 2-h incubation at 37 °C. Absorbance at 450 nm was then measured using a microplate reader, and cell viability was determined using the corresponding calculation formula.

$$\text{Cellviability}\;\left(\%\right)=\frac{\mathrm{OD}\;450\left(\mathrm{Sample}\right)-\mathrm{OD}\;450\left(\mathrm{Mock}\right)}{\mathrm{OD}\;450\left(\mathrm{Mock}\right)-\mathrm{OD}\;450\left(\mathrm{Blank}\right)}\times 100$$ (1)

OD 450 represents the absorbance at 450 nm.

THP-1 cells were seeded in 12-well plates at a density of 5 × 10^5^ cells per well and incubated with PDR as described above. Cells were collected by centrifugation at 300g and stained with Annexin V-FITC/propidium iodide (PI) reagent, and apoptosis was determined by flow cytometry analysis according to the manufacturer’s instructions.

### Animals

The animals were obtained from Sibef Biotechnology Co. Ltd. and housed in the Laboratory Animal Center of Beijing Institute of Technology under specific pathogen-free (SPF) conditions. All animal studies were performed under protocols approved by the Institutional Animal Care and Use Committee (IACUC) of Beijing Institute of Technology, in accordance with institutional ethical guidelines [BIT-EC-SCXK (Beijing) 2024-0001-261].

### Biodistribution of PDR in vivo

Healthy C57BL/6 male mice, 6 to 8 weeks, were fed a standard diet and water for 1 week to acclimatize to the environment. The mice were randomly divided into 3 groups. Naked Cy5-siRNA and PDR_Cy5-siRNA_ were prepared and administered via intravenous injection at a total nucleic acid dose of 1 mg/kg. One group of mice was administered PBS as a negative control. After administration, each mouse was imaged at 1, 3, 6, 12, and 24 h using an IVIS Spectrum imaging system (PerkinElmer, USA) to record fluorescence. Meanwhile, at 3, 6, 12, and 24 h post-injection, one mouse from each group was euthanized, and major organs including the heart, lungs, liver, spleen, kidneys, and bone marrow were harvested for ex vivo fluorescence imaging.

### Antitumor activity of PDR in murine leukemia model

Referring to the reported methods [15], 5 × 10^6^ mouse leukemia cells expressing the human LILRB4 gene (C1498-Luc-hLILRB4) were suspended in PBS (200 μl) and injected into C57BL/6 mice weighing approximately 20 g via the tail vein. After 14 d, mice were randomly divided into 5 groups to receive the following treatments: PBS, DNR, PDR_siNC_, DR_siLILRB4_, and PDR_siLILRB4_. The siRNA dose in groups G3 to G5 was 1 mg/kg. The DNR dose in group G2 was consistent with that in groups G3 to G5, at 2 mg/kg. All groups were treated via tail vein injection, with dosing every 3 d. Treatment was terminated when mice in the PBS group exhibited signs of illness, such as hind-limb paralysis and rapid breathing. Throughout the process, changes in mouse body weight, luciferase signal, and white blood cell (WBC) count in mouse blood were recorded. At the end timepoint, mouse serum was collected, which was sent to DIAN DIAGNOSTICS Group Co. Ltd. for analysis of serum biochemical parameters including aspartate aminotransferase (AST), alanine aminotransferase (ALT), alkaline phosphatase (ALP), urea, lactate dehydrogenase (LDH), and creatine kinase MB (CK-MB). The livers of the mice were then photographed and weighed. Liver tissues were collected and preserved in RNAlater solution for total RNA extraction. The expression level of LILRB4 mRNA was measured by qRT-PCR. Other major organs were fixed in paraformaldehyde, followed by sectioning and analysis using Giemsa, hematoxylin and eosin (H&E), or Ki67 staining. The expression level of LILRB4 protein in liver was detected by Western blot.

### T cell maturation and leukemia cell infiltration analysis

For T cell maturation analysis, after the treatment was concluded, the spleen, lymph nodes, and liver were taken for grinding. After the spleen and lymph nodes were screened with a 40-μm cell strainer and the liver was screened with a 70-μm cell strainer, an appropriate volume of red blood cell cleavage was added. The cells were blocked with 2% bovine serum albumin (BSA) containing Fc blocking reagent and then stained with CD3, CD4, and CD8a fluorescent antibodies. Finally, the CD3^+^, CD4^+^, and CD8^+^ T cell proportions in each tissue were analyzed by flow cytometry.

For leukemia cell infiltration analysis, at the end of the treatment, the content of leukemia cells in blood, liver, and bone marrow was detected. Liver tissue was homogenized, and the resulting cell suspension was passed through a 70-μm filter. For bone marrow, the ends of the femur and tibia were cut off, and PBS was used to flush the bone marrow cavity with a 1-ml injector to collect bone marrow cells. Red blood cells were then removed using lysis buffer. Then, cells were incubated with 2% BSA containing Fc blocking reagent. Subsequently, cells were stained with hLILRB4 (CD85k) fluorescent antibody. Finally, the content of leukemia cells in each tissue was analyzed by flow cytometry.

### Statistical analysis

Statistical analyses were performed using GraphPad Prism 8.0, and data are presented as mean ± SD. For comparisons among multiple groups, one-way analysis of variance (ANOVA) followed by Tukey’s post hoc tests were employed. Differences between 2 groups were assessed using a 2-tailed Student’s *t* test. A *P* value of less than 0.05 was considered statistically significant (*P < 0.05, **P < 0.01, ***P < 0.001, ****P < 0.0001).

## Results and Discussion

### Characterization of PDR

To enhance the endosomal escape efficiency of the carrier-free system, DNR, a first-line chemotherapeutic agent for AML, was covalently conjugated to PEG-2000 through a pH-responsive hydrazone bond to form a DNR prodrug. Fig. S2 showed the synthetic route of the DNR prodrug and its structural confirmation by ^1^H nuclear magnetic resonance (NMR).

**Fig. 2.**
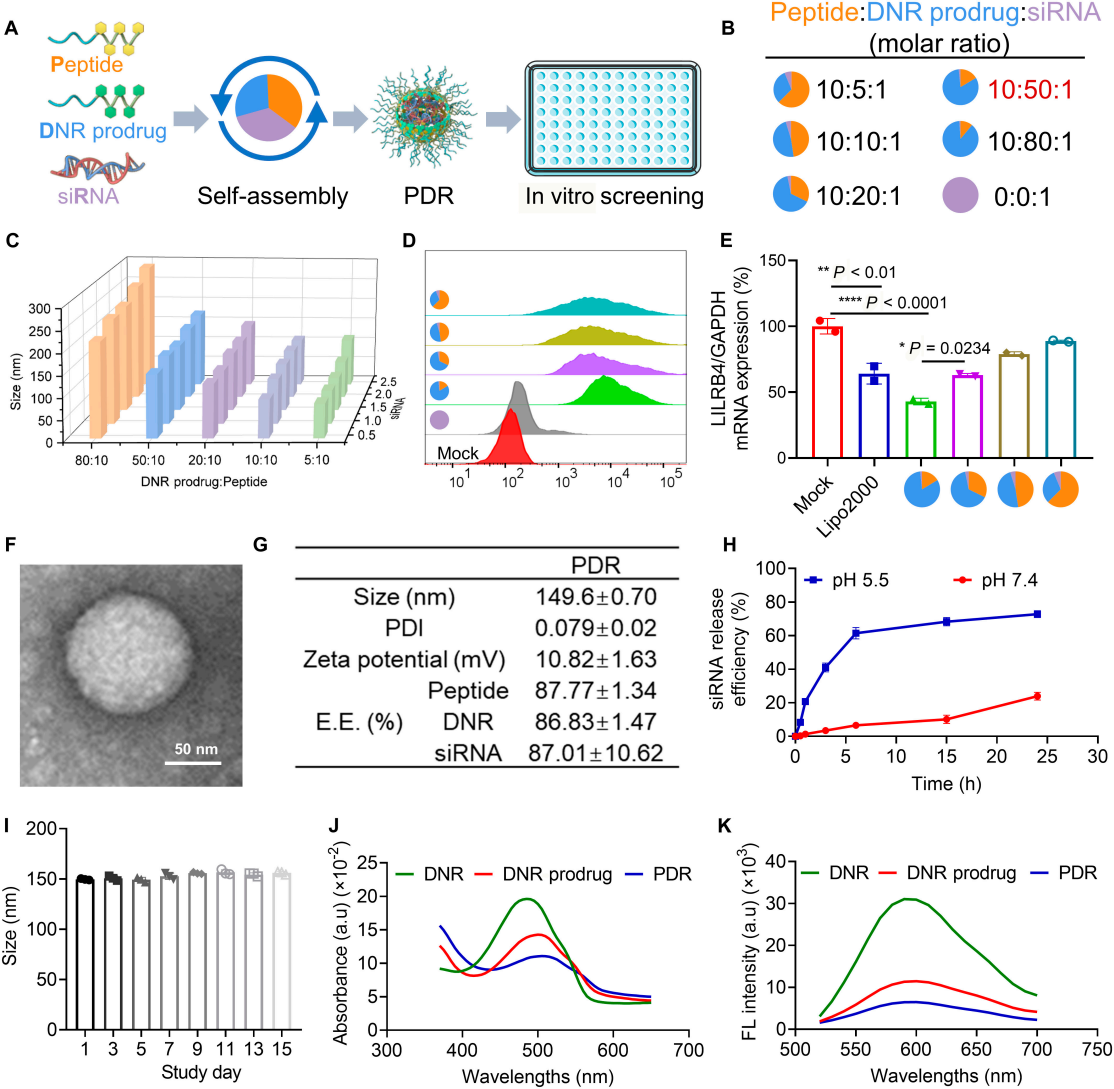
Optimization of the PDR formulation. (A and B) Preparation process of the self-assembly of peptide, DNR prodrug, and siRNA, along with the corresponding formulation ratios. (C) DLS detection of the particle size of PDR at different ratios. (D) Flow cytometry detection of the uptake efficiency of THP-1 cells for PDR at different molar ratios. Cells were transfected with 100 nM siRNA for 4 h. (E) RT-qPCR detection of gene inhibition efficiency in THP-1 cells 24 h after transfection with PDR at various ratios (*n* = 2). (F) TEM image showing the morphology of PDR nanoassembly (peptide:DNR-progrug:siRNA at 10:50:1, the final formulation). Scale bar, 50 nm. (G) Hydrodynamic size, polydispersity index (PDI), zeta potential, and encapsulation efficiency of PDR nanoassembly. (H) Release efficiency of PDR in PBS buffer at pH 5.5 and pH 7.4. (I) Evaluation of PDR stability by detecting changes in particle size at different times (*n* = 3). (J and K) The presence of π–π stacking interactions in PDR was confirmed by comparing UV absorption wavelengths (J) and fluorescence intensities (K) before and after self-assembly. The concentration of siRNA was 100 nM. All characterization data in (F) to (K) correspond to the final formulation of the PDR nanoassembly at a peptide:DNR-prodrug:siRNA ratio of 10:50:1. *P < 0.05, **P < 0.01, ****P < 0.0001. *P* value determined by one-way ANOVA followed by Tukey’s multiple comparisons test.

The peptide, DNR prodrug, and siRNA were mixed at various molar ratios and self-assembled into PDR nanoparticles, followed by in vitro experiments to determine the optimal formulation (Fig. 2A). The siRNA loading efficacy of the peptide was evaluated. The agarose gel electrophoresis results showed that siRNA almost completely complexed at a peptide-to-siRNA molar ratio of 10:1 (Fig. S3A). The proportion of the DNR prodrug was gradually increased (Fig. 2B), and the resulting nanoparticles were systematically evaluated in terms of particle size, siRNA loading efficiency, cellular uptake efficiency, and gene silencing efficiency. These parameters were assessed using DLS, agarose gel electrophoresis, flow cytometry (FACS), CLSM, and real-time qPCR, respectively. DLS analysis revealed that the particle size of PDR increased progressively with the rising proportion of the DNR prodrug. We propose that this phenomenon may be attributed to the increased proportion of the DNR prodrug, which enhances the hydrophobic interactions and π–π stacking effects between the hydrophobic DNR molecules, peptides, and siRNA. As a result, the nanoparticle core becomes more compact, while the tendency for particle aggregation may also increase, ultimately leading to a larger particle size of the PDR as the proportion of the DNR prodrug rises. When molar ratio (peptide:DNR-progrug:siRNA) was 10:80:1, the particle size of PDR exceeded 200 nm (Fig. 2C) and was thus excluded from candidate formulations. Agarose gel electrophoresis results showed that siRNA was almost completely loaded across all tested ratios (Fig. S3B). The candidate formulation was transfected into THP-1 cells at a final siRNA concentration of 100 nM. The cellular uptake efficiency of preparations with different drug ratios was evaluated using FACS and CLSM, while the gene silencing effect on the target gene was detected by RT-qPCR. The results demonstrated that as the ratio of DNR prodrug increased, both the cellular uptake efficiency and gene silencing efficiency of the preparations were progressively enhanced (Fig. 2D and E and Fig. S3C). We propose that the enhanced cellular uptake efficiency of the PDR formulation is primarily attributed to the increased proportion of the DNR prodrug, which elevates the surface charge of PDR (Fig. S4A), thereby strengthening the electrostatic interaction between PDR and the negatively charged cell membranes and facilitating endocytosis. Meanwhile, the improvement in gene silencing efficiency results from the synergistic effects of 2 factors. First, the increased proportion of the DNR prodrug enhances cellular uptake of PDR. Second, a higher DNR prodrug ratio introduces more pH-sensitive hydrazone bonds into the PDR formulation, enabling faster degradation in the acidic endosomal environment and promoting the release of siRNA into the cytoplasm, ultimately improving gene silencing efficiency. Considering all the above factors, a molar ratio of peptide:DNR-progrug:siRNA at 10:50:1 was ultimately selected as the optimal formulation for PDR.

TEM revealed that the optimized PDR nanoparticles were spherical in shape (Fig. 2F), while DLS results showed that the particle size was approximately 150 nm, with a polydispersity index (PDI) of about 0.079 and a zeta potential of around 10.82 mV (Fig. 2G and and Fig. S4B). Furthermore, we conducted a systematic quantitative analysis of the encapsulation efficiency of siRNA, the DNR-prodrug, and the functionalized peptide in the PDR formulation. The results demonstrated that the encapsulation efficiencies of siRNA, DNR-prodrug, and functionalized peptide in the formulation were 87.01%, 86.83%, and 87.77%, respectively (Fig. 2G). In vitro siRNA release experiment results showed that PDR quickly disintegrated and released siRNA under acidic conditions (pH 5.5), reaching a maximum release rate of ~70% within 6 h. In contrast, siRNA release was markedly slower at physiological conditions (pH 7.4), with only ~20% released over 24 h (Fig. 2H). This demonstrates that PDR is capable of endosomal escape under the mildly acidic conditions of lysosomes and endosomes. In addition, monitoring of particle size changes revealed that PDR remained stable for at least 2 weeks (Fig. 2I). To further evaluate the physiological stability of PDR, we monitored changes in the particle size of PDR nanoparticles incubated in saline and 10% FBS (to simulate a serum environment) at 37 °C. The experimental results showed that in saline, the particle size of PDR nanoparticles remained stable for up to 7 d without significant changes (Fig. S5A). In the solution containing 10% FBS, the particle size of PDR gradually increased over time, exceeding 300 nm by day 6, after which it rose sharply (Fig. S5B). In summary, PDR nanoparticles remained stable for at least 7 d in saline and for 5 d in 10% FBS, with noticeable degradation occurring from day 6 onward. These results demonstrate the potential of the PDR formulation for in vivo applications and provide an experimental basis for selecting the administration window in subsequent animal studies.

The occurrence of intermolecular π–π stacking interactions within the PDR system was further supported by changes in its optical properties. Specifically, a distinct redshift in the UV–vis absorption spectrum was observed, with the maximum absorption peak shifting from 485 nm (free DNR) to 510 nm (assembled PDR) (Fig. 2J). This bathochromic shift is indicative of enhanced π–electron delocalization, commonly associated with π–π stacking among aromatic groups during the self-assembly process. In addition, a marked decrease in fluorescence intensity was observed following nanoparticle formation (6,830), compared to both free DNR (33,538) and the DNR prodrug (12,161) (Fig. 2K). This fluorescence quenching effect is consistent with the proximity of aromatic moieties and the formation of nonradiative energy dissipation pathways, which are characteristic features of π–π stacking. Collectively, these spectral changes provide indirect but compelling evidence that π–π interactions play a critical role in the self-assembly and structural stabilization of the PDR nanoparticles.

### Internalization behavior and endosomal escape of PDR

To verify the cellular uptake efficiency of PDR, Cy5-labeled siRNA was loaded into PDR (PDR_siCy5_) and the internalization behavior of PDR was assessed in THP-1 cells using FACS.

**Fig. 3.**
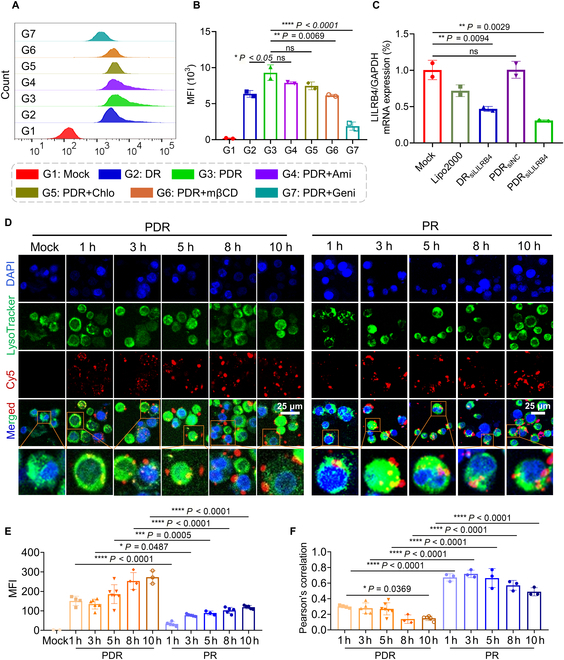
Internalization behavior and endosomal escape of PDR. (A) Evaluation of PDR endocytosis efficiency and exploration of endocytosis mechanisms. Flow cytometry analysis was performed on THP-1 cells following a 4-h transfection period with siRNA at a concentration of 100 nM for each group. DR is the self-assembled nanoparticle without targeting transmembrane peptide. Ami, amiloride hydrochloride, inhibits macropinocytosis; Chlo, chlorpromazine hydrochloride, inhibits clathrin-mediated endocytosis; mβCD, methyl-β-cyclodextrin, inhibits caveolae-mediated endocytosis; Geni, genistein, inhibits caveolin-mediated endocytosis. (B) Quantification analysis of (A) (*n* = 2). (C) RT-qPCR assessment of gene silencing efficiency in THP-1 cells following 24-h transfection with Lipo2000_siLILRB4_, DR_siLILRB4_, PDR_siNC_, and PDR_siLILRB4_ (*n* = 2). siRNA was transfected at a concentration of 100 nM. (D) Endosomal efficiency evaluation of PDR and PR. Scale bars, 25 μm. PR is the targeted self-assembled nanoparticle without pH-responsive DNR prodrug. Nucleus, endosome/lysosome, and siRNA were stained with 4′,6-diamidino-2-phenylindole (DAPI) (blue), LysoTracker Green (green), and Cy5 (red), respectively. (E) Quantification analysis of the average Cy5 fluorescence intensity of PDR and PR shown in (D). (F) Colocalization analysis of Cy5 signal with lysosome signal of PDR and PR shown in (D). The smaller the Pearson’s correlation value, the less overlapped fluorescence signal, indicating higher endosomal escape efficiency. *P < 0.05; **P < 0.01; ****P < 0.0001; ns, not significant difference. *P* value determined by one-way ANOVA followed by Tukey’s multiple comparisons test.

To explore the cellular uptake mechanism of PDR, methyl-β-cyclodextrin, amiloride hydrochloride, chlorpromazine hydrochloride, and genistein were employed to inhibit the endocytosis mediated by lipid raft, macropinocytosis, clathrin, and caveolin, respectively. After incubating with the above 4 inhibitors for 30 min, THP-1 cells were further incubated with PDR_siCy5_ for 4 h at 37 °C in the absence of light conditions, followed by FACS assessment of internalization efficiency. The results showed that, compared to DR (daunorubicin-siRNA, which does not contain peptide and is assembled from DNR prodrug and siRNA), which lacks peptide-mediated targeting capability, PDR exhibited significantly enhanced cellular uptake efficiency (Fig. 3A and B), suggesting that the incorporation of the functionalized cell-penetrating peptide (CPP44-p16^MIS^-FFY-4R) contributed to the improved internalization of the PDR system. The inhibitory effect on the target gene of LILRB4 was confirmed by Western blot and qPCR. The results were consistent with FACS analysis, indicating that PDR has a better inhibitory effect on the target gene than DR (Fig. 3C and Fig. S6). The inhibitor experiment results showed that genistein had the greatest impact on the internalization of PDR, followed by methyl-β-cyclodextrin, while chlorpromazine and amiloride had little effect on the internalization of PDR, suggesting that the cellular uptake of PDR mainly depends on caveolin and is less dependent on lipid rafts, and hardly depends on clathrin and macropinocytosis (Fig. 3A and B).

To further assess the endosomal escape effect mediated by DNR prodrug, the internalization of Cy5-siRNA and its colocalization with lysosomes were observed by CLSM after transfecting THP-1 cells with PDR and PR (peptide-siRNA, which does not contain DNR prodrug and is assembled from peptides and siRNA) for 1, 3, 5, 8, and 10 h. The results showed a time-dependent increase in intracellular Cy5 fluorescence intensity following transfection with both PDR and PR (Fig. 3D, E, and G), indicating that both PDR and PR could enter THP-1 cells through endocytosis. Additionally, Pearson’s correlation analysis showed that the colocalization between siRNA and endosomes peaked at 3 h post-transfection with PDR and subsequently declined. In contrast, in cells transfected with PR, the colocalization signal remained relatively stable and only began to decrease after 8 h (Fig. 3D, F, and H). These results indicate that the introduction of the hydrazone-linked DNR prodrug effectively facilitates endosomal escape.

### In vitro efficacy and mechanism studies of PDR

**Fig. 4.**
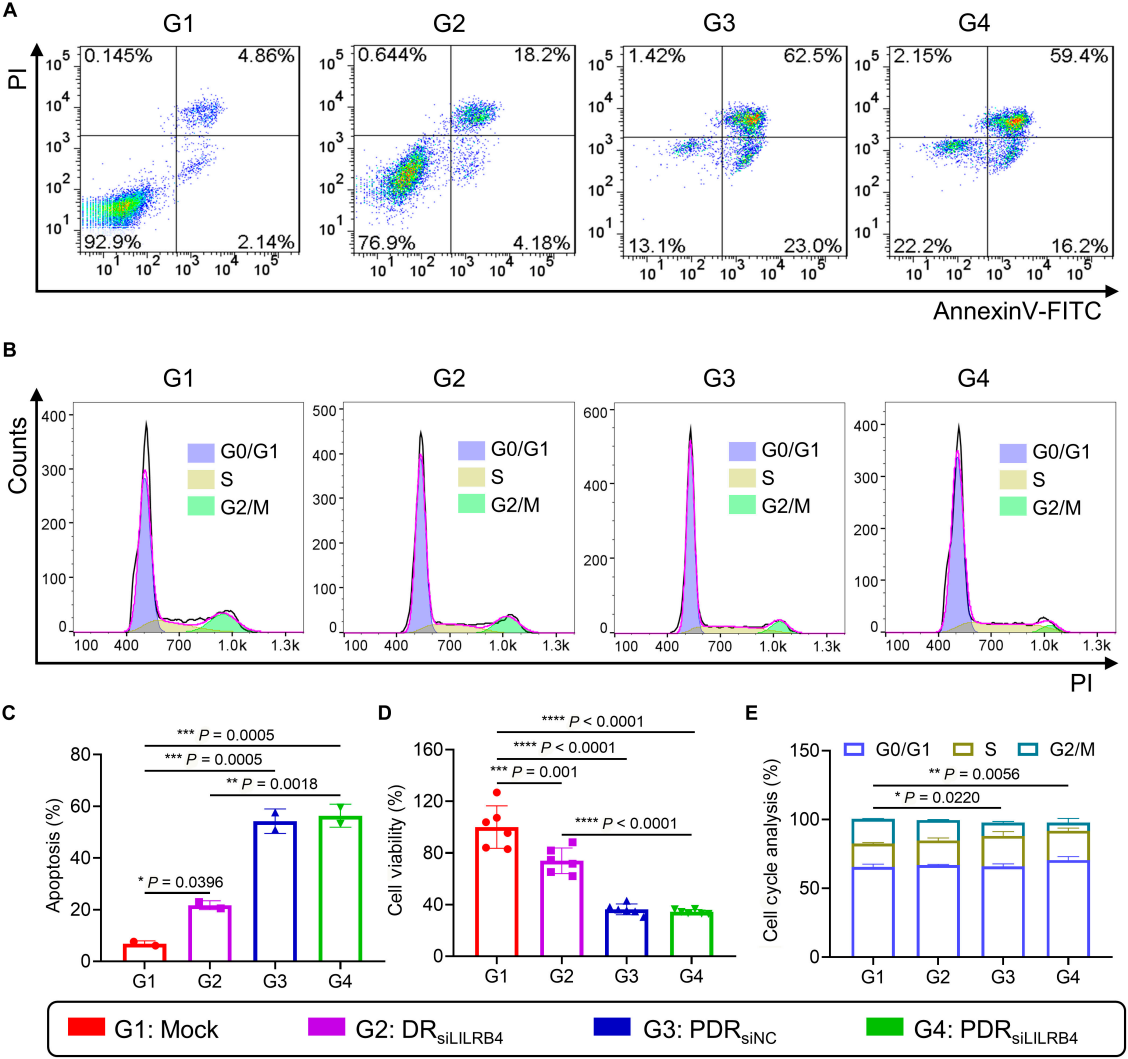
In vitro efficacy and mechanism studies of PDR. (A) FACS assessment of the apoptosis efficiency of PDR on THP-1 cells. (B) The cell cycle was analyzed by flow cytometry. (C) Quantitative analysis of the cell apoptosis was performed (*n* = 2). (D) Cell viability of THP-1 cell-treated PDR was assessed using the CCK-8 assay (*n* = 6). (E) Quantitative analysis of cell cycle (*n* = 2). A transfection concentration of 100 nM siRNA was used in all cellular assays. The *P* value represents the result of the statistical analysis of the G2/M phase. For the apoptosis assay, cell cycle analysis, and CCK-8 assay, THP-1 cells were transfected with siRNA at a concentration of 100 nM for 24 h. *P < 0.05; **P < 0.01; ***P < 0.001; ****P < 0.0001. *P* value determined by one-way ANOVA followed by Tukey’s multiple comparisons test.

To verify the cytotoxicity of PDR against AML cells, PDR was added into THP-1 cell and incubated for 24 h, followed by analysis of apoptosis induction and proliferation inhibition using FACS and the CCK-8 assay, respectively. FACS results showed that the apoptosis efficiency mediated by PDR was approximately 60% (Fig. 4A and C), and CCK-8 results indicated that the proliferation inhibition efficiency of PDR was about 65% (Fig. 4D). Additionally, results from both flow cytometry and CCK-8 assays indicated that there were no significant differences in cytotoxicity between PDR_siLILRB4_ and PDR_siNC_ toward leukemia cells, suggesting that inhibition of LILRB4 gene expression did not affect the viability of leukemia cells. This finding is consistent with previous studies [15,16], which reported that the LILRB4 gene is primarily involved in the infiltration of leukemia cells and the suppression of T cell function, rather than directly regulating the proliferation of leukemia cells.

To further verify the effect of the p16^MIS^ sequence on the cell cycle of leukemia cells, THP-1 cells were incubated with PDR for 24 h, followed by cell cycle analysis using FACS. Compared to the DR_siLILRB4_ treatment group lacking the p16^MIS^ sequence, the PDR_siLILRB4_ and PDR_siNC_ treatment groups containing the p16^MIS^ sequence exhibited a substantial decline in the proportion of G2/M phase (Fig. 4B and E). This suggests that p16^MIS^ effectively inhibits intracellular DNA replication, thereby blocking the cell transition from phase of S to G2/M and inducing apoptosis.

### In vivo biodistribution of PDR NPs

To evaluate the organ distribution characteristics of the PDR nanoassembly in vivo and its ability to avoid rapid clearance, we conducted an in vivo distribution study. The PDR nanoassembly was administered via tail vein injection to C57BL/6 mice at a dose equivalent to 1 mg/kg of siRNA, and its distribution in the heart, liver, spleen, lungs, kidneys, and bone marrow was monitored over a 24-h period. The experiment included 3 groups: group G1 received PBS as a control, group G2 was injected with naked Cy5-siRNA, and group G3 received Cy5-labeled PDR nanoassembly (PDR_Cy5-siRNA_). Imaging results revealed that PDRsiLILRB4 primarily accumulated in the spleen and liver, consistent with the typical distribution pattern of nanocarriers mediated by the enhanced permeability and retention (EPR) effect and uptake by the mononuclear phagocyte system. Additionally, significant signals were detected in the kidneys, which align with the physiological role of this organ as a major pathway for drug metabolism and excretion (Fig. S7A to F). Of particular note, compared to the free siRNA group, the PDR formulation exhibited a significantly stronger fluorescence signal in the bone marrow (Fig. S7G). This finding provides experimental evidence supporting the potential of the PDR system to reduce the infiltration of leukemia cells into the bone marrow. Further quantitative analysis of the distribution of PDR in various organs showed that at the 3-h time point post-injection, the distribution percentages were 19.59% in the liver, 28.67% in the spleen, and 40.07% in the kidneys (Fig. S7H). These in vivo distribution results provide important evidence for further exploration of the PDR nanoassembly as a therapeutic strategy for AML.

### In vivo treatment efficacy of PDR

**Fig. 5.**
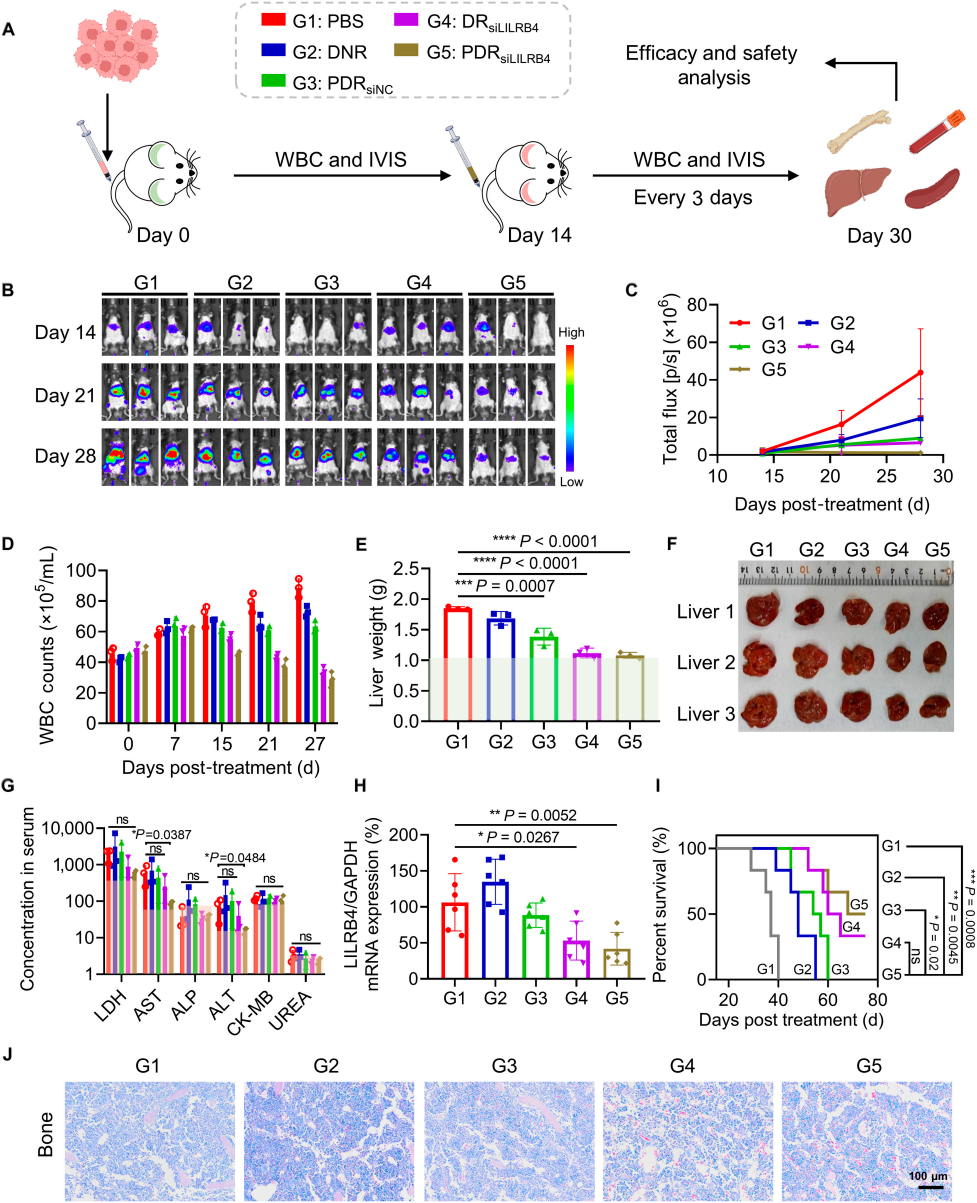
In vivo anti-leukemia efficacy of PDR. (A) Dosing plan and subject grouping overview. (B) Bioluminescence imaging of mice at specific times following different treatments. d-Luciferin was injected intraperitoneally (100 μl of a 10 mg/ml solution) 10 min before in vivo imaging. (C) Quantification analysis of (B). (D) White blood cell (WBC) number of mouse blood at specific times after different treatments (*n* = 3). (E) Liver weights of mice at the end time point (*n* = 3). The gray part represents the normal liver weight of the mouse. *P* value determined by one-way ANOVA followed by Tukey’s multiple comparisons test. (F) Representative images of the livers of mice receiving different treatments at the end of treatment. (G) Six serum biochemical indicators were detected, including alanine aminotransferase (ALT, U/l), alkaline phosphatase (ALP, U/l), creatine kinase MB (CK-MB, U/l), lactate dehydrogenase (LDH, U/l), aspartate aminotransferase (AST, U/l), and serum creatinine (CREA, μM) (*n* = 3). The gray parts represent the normal levels of the indicators. (H) Expression of hLILRB4 mRNA in mouse liver. (I) Survival curves of mice (*n* = 6). Survival curves are plotted using the Kaplan–Meier method and compared using the log-rank test. (J) Representative images of Giemsa staining of mouse femurs at the end of treatment. The normal ranges of the indicators are shown as shaded areas. *P < 0.05; **P < 0.01;***P < 0.001; ****P < 0.0001.

To further assess the treatment outcomes of PDR, a mouse C1498-Luc cell line stably expressing the human LILRB4 gene (C1498-Luc-hLILRB4) was established. Subsequently, leukemia mouse models were established by intravenously injecting 5 × 10^6^ C1498-Luc-hLILRB4 cells into each mouse. After 14 d, mice were randomly divided into 5 groups for PDR administration, which was carried out every 3 d with an siRNA dosage of 1 mg/kg each time. On day 30, the mice were euthanized for analysis (Fig. 5A). Body weight of the mice was monitored throughout the treatment period, and bioluminescence imaging and WBC counts were performed to monitor the progression of leukemia in the mice. No considerable decrease in body weight of other groups was observed, except for the DNR treatment group that showed a slight decrease in body weight after administration, indicating favorable in vivo safety profile of PDR (Fig. S8A). The results of bioluminescence imaging and WBC counts showed that the progression of leukemia was greatly delayed after PDR treatment (Fig. 5B to D). Bioluminescence imaging results showed that leukemia cells primarily infiltrated the liver of mice, suggesting that the extent of liver lesions may serve as a critical indicator for evaluating drug treatment efficacy. Therefore, after dissection, liver size and weight were examined. The results showed that PDR treatment significantly reduced liver enlargement and restored liver weight to normal levels (Fig. 5E and F). Serum biochemical tests showed that after the onset of leukemia, the expression levels of multiple liver and kidney indicators significantly increased, but after PDR drug treatment, all indicator levels returned to normal (Fig. 5G).

In addition, qPCR and Western blot detection of liver tissue showed that PDR treatment effectively mediated target gene silencing in vivo. Compared to the DR group lacking the cell-penetrating peptide, PDR exhibited a stronger inhibitory effect on the target gene, highlighting the critical role of the CPP44-targeting cell-penetrating peptide in enhancing in vivo delivery efficiency (Fig. 5H and Fig. S8B). At the same time, PDR treatment could significantly extend the survival period of leukemia model mice (Fig. 5I). Notably, compared to G2 (treated with DNR alone), the significantly prolonged survival of mice in G5 (treated with PDR_siLILRB4_) highlights the synergistic effect of combining chemotherapy with immunotherapy. Furthermore, the markedly extended survival observed in G5 relative to G3 (treated with PDR_siNC_) underscores the critical role of inhibiting LILRB4 expression in achieving the therapeutic outcome. Subsequently, Giemsa staining on the bone marrow tissues of mice was performed. Since red blood cells lack a nucleus, they can be stained pink, while other WBCs are easily stained blue by Giemsa stain. WBC content of bone marrow can be distinguished based on the blue and pink colors. According to the staining results (Fig. 5J), it was observed that the bone marrow cells in the PBS group were almost entirely blue, with extremely low red blood cell content. In contrast, the bone marrow cells of the PDR-treated mice exhibited a significant increase in red blood cell content, indicating that the PDR system effectively reduced leukemia cell infiltration.

**Fig. 6.**
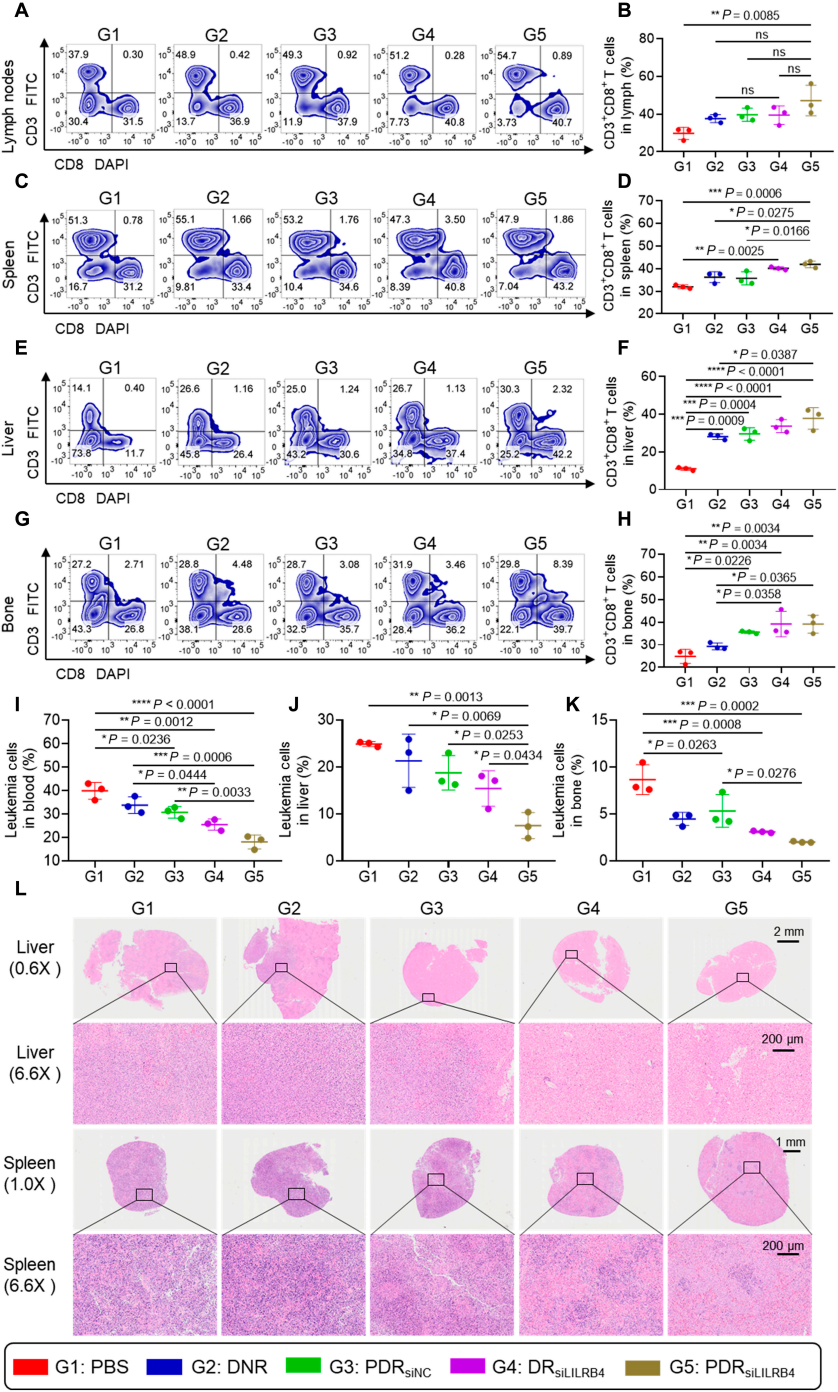
Evaluation of T cell differentiation and leukemic cell infiltration. (A) FACS determination of T cell maturation in lymph nodes. (B) Quantification analysis of CD3^+^ CD8^+^ cell in (A) (*n* = 3). (C) FACS determination of T cell maturation in spleen. (D) Quantification analysis of CD3^+^ CD8^+^ cell in (C) (*n* = 3). (E) FACS determination of T cell maturation in liver. (F) Quantification of CD3^+^ CD8^+^ cell in (E) (*n* = 3). (G) FACS determination of T cell maturation in bone marrow. (H) Quantification of CD3^+^ CD8^+^ cell in (G) (*n* = 3). (I to K) At the end of treatment, the leukemia cell infiltration in blood (I), liver (J), and bone (K) of mice receiving different therapies (*n* = 3). (L) Histological analysis of liver and spleen tissues by H&E staining. *P < 0.05; **P < 0.01;***P < 0.001; ****P < 0.0001. *P* value determined by one-way ANOVA followed by Tukey’s multiple comparisons test.

Studies have shown that the LILRB4-related pathway mediates T cell suppression and tumor infiltration [15]. To verify the function of anti-LILRB4 siRNA, T cell maturation by detecting the proportion of CD3^+^ CD8^+^ T cells in the spleen and lymph nodes of mice were assessed via flow cytometry after treatment. It was found that after PDR_siLILRB4_ treatment, the proportion of CD3^+^ CD8^+^ T cells in the lymph nodes of mice significantly increased (Fig. 6A and B). Similarly, after PDR_siLILRB4_ and DR_siLILRB4_ treatments, the CD3^+^ CD8^+^ T cell proportion of spleen also significantly increased (Fig. 6C and D). However, no marked changes of CD3^+^ CD8^+^ T cell proportion in the lymph nodes and spleen were observed compared to the PBS group after treatment with DNR without siRNA or PDR_siNC_ containing nonsense siRNA. The CD3^+^ CD8^+^ T cell proportion in both bone marrow and liver (the main organs infiltrated by leukemia) were also detected by FACS, and the results were consistent with the above in spleen and lymph nodes (Fig. 6E to H). This indicates that silencing the expression of the LILRB4 gene in leukemia cells not only promotes T cell maturation in the lymph nodes and spleen but also enhances T cell infiltration into both primary and infiltrated leukemia tissues.

The extent of leukemia cell infiltration in various organs was evaluated by quantifying the proportion of hLILRB4^+^ cells in the blood, liver, and bone marrow of mice. It was found that after PDR_siLILRB4_ treatment, the hLILRB4^+^ proportion in the blood, liver, and bone marrow of mice was significantly decreased (Fig. 6I to K and Fig. S9). It is noteworthy that, compared to G4 (treated with DR_siLILRB4_), mice in group G5 (treated with PDR_siLILRB4_) exhibited a significant reduction in leukemic cell infiltration in the liver. This result demonstrates that the peptide component plays a crucial role in guiding specific drug targeting and enhancing the anti-leukemic effect, further supporting the therapeutic potential of the PDR system in suppressing leukemia-related organ infiltration. Furthermore, H&E staining results revealed extensive and dense leukemic cell infiltration in the liver and spleen of PBS-treated mice, accompanied by disorganized tissue architecture and significant pathological damage. Focal mild infiltration was also observed in the lungs, while the heart and kidney tissues remained largely unaffected, indicating a homing tendency of leukemia cells to specific organs. In contrast, treatment with PDR_siLILRB4_ significantly reduced both the area and density of infiltration in the liver and spleen, improved the integrity of the tissue structure, and markedly alleviated the extent of damage. These findings demonstrate that PDR_siLILRB4_ not only effectively reduces systemic leukemia burden but also strongly inhibits the migration and homing of leukemic cells to key immune and metabolic organs such as the liver and spleen. Given that the liver and spleen are major sites for the proliferation, accumulation, and immune escape of myeloid leukemia cells, the pronounced reduction in infiltration following LILRB4-targeted intervention highlights the therapeutic potential of blocking the LILRB4 signaling pathway to disrupt the leukemia-supportive microenvironment. This effect may be attributed to both the direct cytotoxic impact of PDR_siLILRB4_ on leukemic cells and the enhanced recruitment or activation of anti-leukemic immune responses through modulation of the tumor immune microenvironment (Fig. 6L and Fig. S10A). The Ki67 staining results of the liver and spleen were consistent with the H&E findings, further supporting the observed reduction in leukemic cell proliferation following PDR_siLILRB4_ treatment (Fig. S10B). As Ki67 is a well-established marker of cell proliferation, its down-regulation further supports the conclusion that PDR_siLILRB4_ effectively suppresses leukemic cell expansion in infiltrated tissues. This reduced proliferative index, in combination with decreased leukemic infiltration observed via H&E staining, suggests that the therapeutic efficacy of PDR_siLILRB4_ may stem not only from cytotoxic effects but also from disruption of the local microenvironment that supports leukemia cell survival and proliferation. Moreover, the dual impact on infiltration and proliferation highlights the potential of LILRB4-targeted RNA interference (RNAi) strategies to remodel leukemic niches and achieve broader disease control. These results indicate that siRNA-mediated gene silencing of LILRB4 in leukemia cells can effectively promote T cell maturation and inhibit leukemia infiltration of various organs.

## Conclusion

In this study, we successfully developed a self-assembled, carrier-free nanotherapeutic system—PDR—composed of a multifunctional peptide, a pH-responsive DNR prodrug, and siRNA targeting LILRB4. This platform integrates targeted delivery, chemotherapy, and gene silencing to achieve multi-mechanistic anti-leukemic effects. The peptide component not only enables selective recognition and internalization into AML cells via the CPP44 domain but also delivers the tumor-suppressive p16^MIS^ sequence, which induces phase arrest and apoptosis by disrupting cell cycle progression. Upon endocytosis, the hydrazone bond in the DNR prodrug is cleaved in the acidic endosomal environment, facilitating both endosomal escape and intracellular drug release. Concurrently, siRNA-mediated silencing of LILRB4 reduces leukemic cell infiltration into major organs and enhances T cell maturation, contributing to an immune-mediated anti-leukemic response. Collectively, the PDR nanodrug demonstrates potent anti-AML efficacy by combining cell cycle interference, chemotherapeutic cytotoxicity, and immunomodulation, demonstrating its strong potential for clinical translation in AML therapy.

## Data Availability

The data and materials used to support the findings of this study are available from the corresponding author upon reasonable request.
