# Supplementary material for: Carrier-Free Peptide–Daunorubicin–Small Interfering RNA Nanoassembly for Targeted Therapy of Acute Myeloid Leukemia
Source: Cyborg Bionic Syst. 2025 Nov 5;6:0436. doi: 10.34133/cbsystems.0436 (PMC12586850; doi:10.34133/cbsystems.0436)
Supplement: Supplementary 1 — Figs. S1 to S10 [file cbsystems.0436.f1.docx]

**Supplementary Materials**

**Carrier-free Peptide-Daunorubicin-Small Interfering RNA Nanoassembly for Targeted Therapy of Acute Myeloid Leukemia**

Haiyin Yang ^1^, Xi Yu ^1^, Zhitong Guo ^1^, Songxuan Shi ^1^, Jie Wang ^1^, Shuai Guo ^1^, Bo Hu ^1^, Meihong Chai ^2,3^, Zhuoran Wang ^4^, Stefan Barth ^5^, Kelong Fan ^4^, Huining He ^3,^*, Mengjie Zhang ^1,6,7,^*, Yuanyu Huang ^1,5,7,^*

^1^ School of Life Science; School of Interdisciplinary Science; Aerospace Center Hospital; Key Laboratory of Molecular Medicine and Biotherapy; Key Laboratory of Medical Molecule Science and Pharmaceutics Engineering; Beijing Institute of Technology, Beijing 100081, P. R. China.

^2^ Tianjin Key Laboratory on Technologies Enabling Development of Clinical Therapeutics and Diagnostics, School of Pharmacy, Tianjin Medical University, Tianjin 300070, P. R. China.

^3^ Xi'an Hospital of Traditional Chinese Medicine, Xi'an, 710021, P. R. China.

^4^ CAS Engineering Laboratory for Nanozyme, Key Laboratory of Protein and Peptide Pharmaceutical, Institute of Biophysics, Chinese Academy of Sciences, Beijing 100101, P. R. China.

^5^ South African Research Chair in Cancer Biotechnology, Institute of Infectious Disease and Molecular Medicine (IDM), Department of Integrative Biomedical Sciences, Faculty of Health Sciences, University of Cape Town, Cape Town 7925, South Africa.

^6^ School of Medical Engineering; School of Interdisciplinary Science; Affiliated Zhuhai People's Hospital; Beijing Institute of Technology, Zhuhai, 519088, P. R. China.

^7^ Advanced Technology Research Institute, Beijing Institute of Technology, Ji’nan, 250100, P. R. China.

* Correspondence should be addressed to Yuanyu Huang; [yyhuang@bit.edu.cn](mailto:yyhuang@bit.edu.cn) and Mengjie Zhang; [zmj@bit.edu.cn](mailto:zmj@bit.edu.cn) and Huining He; [hehuining@tmu.edu.cn](mailto:hehuining@tmu.edu.cn)


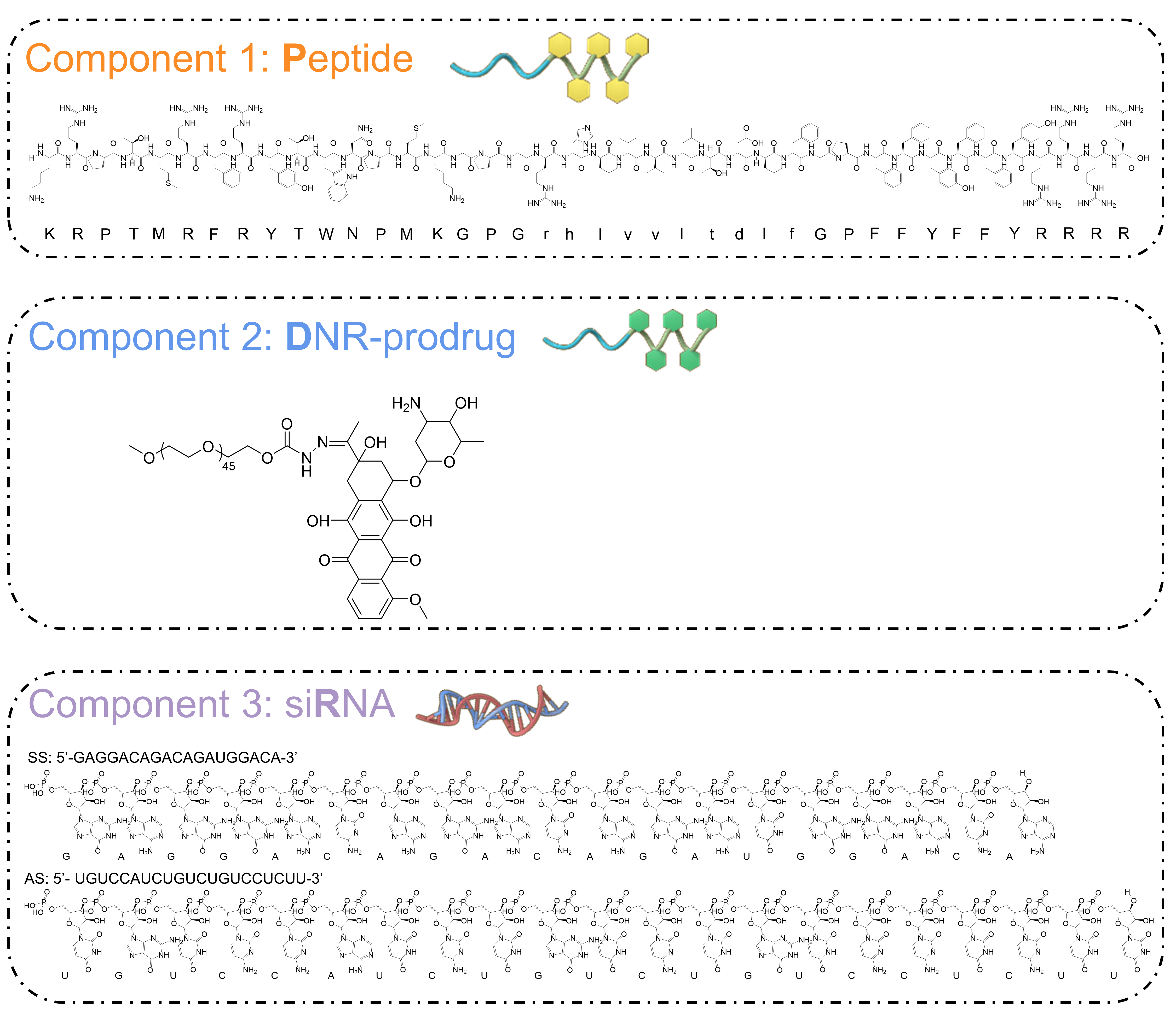


**Figure S1.** Chemical structures of the three functional components constituting the PDR nanoassembly: Peptide (KRPTMRFRYTWNPMK-GPG-rhlvvltdlf-GP-FFYFFY-RRRR); DNR-prodrug; siRNA (SS: 5’-GAGGACAGACAGAUGGACA-3’, AS: 5’- UGUCCAUCUGUCUGUCCUCUU-3’).

**
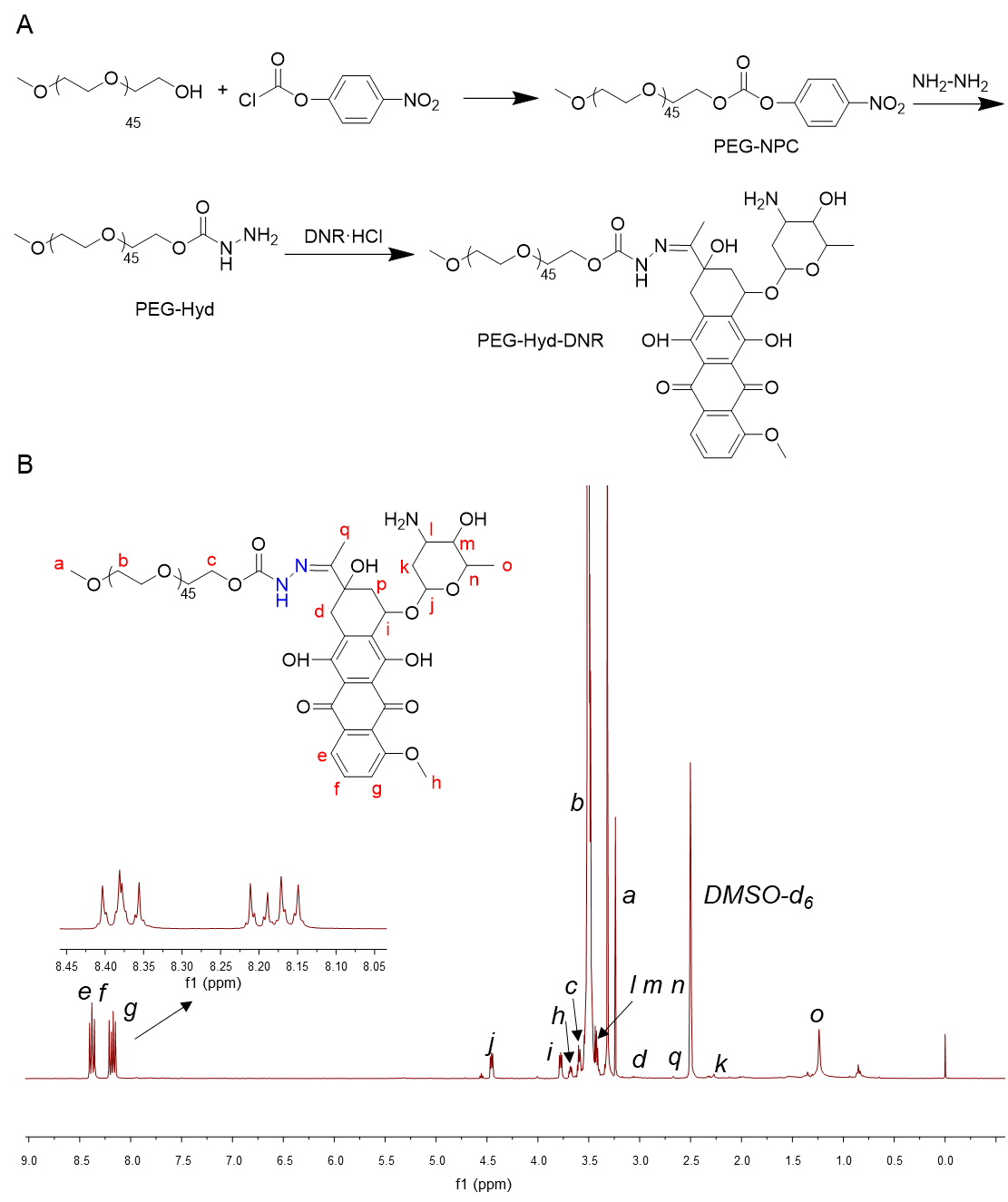
**

**Figure S2.** A) The synthesis route of DNR-produrg. B) ^1^H NMR spectrum of DNR-prodrug (in DMSO-d6).

**
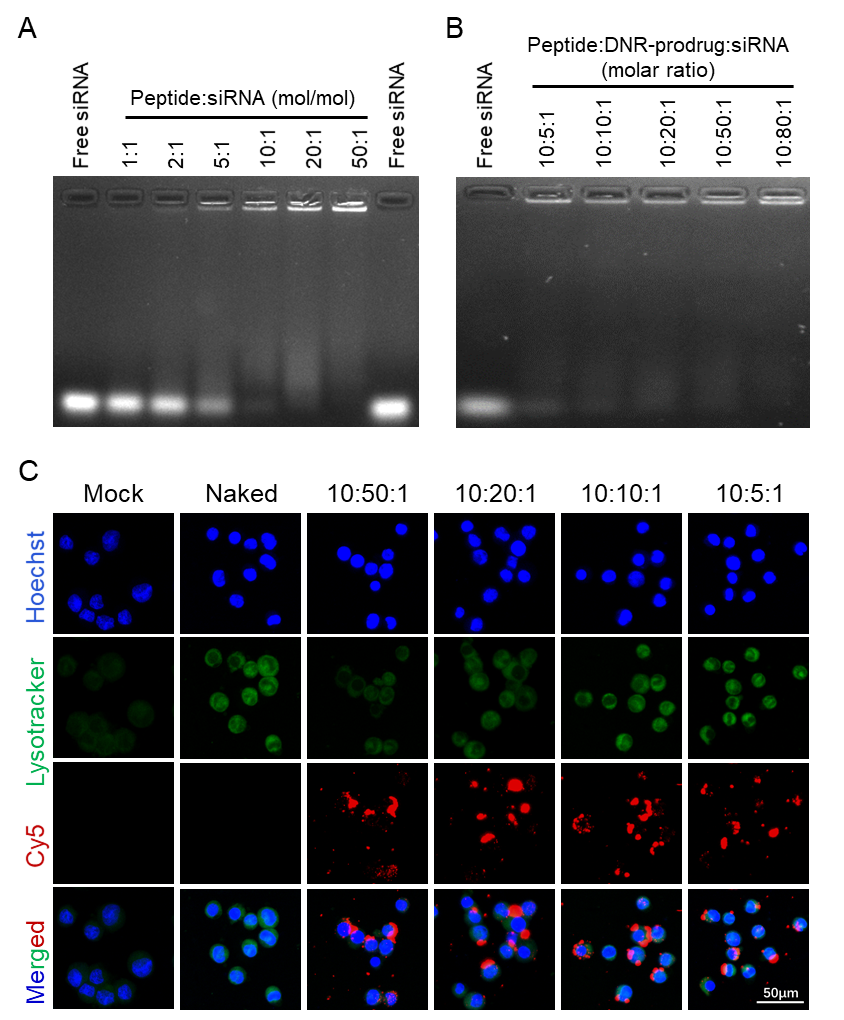
**

**Figure S3.** Optimization and characterization of PDR. A), B) The loading efficiency of siRNA at different ratios was analyzed by agarose gel electrophoresis. C) Confocal laser scanning microscopy (CLSM) was used to evaluate the uptake efficiency of THP-1 cells toward PDR with different component ratios. Free (naked) siRNA was used as a negative control. siRNA was transfected at concentration of 100 nM. Nucleus, endosome/lysosome, and siRNA were stained with Hoechst 33342 (blue), Lysotracker Green (green), and Cy5 (red), respectively.


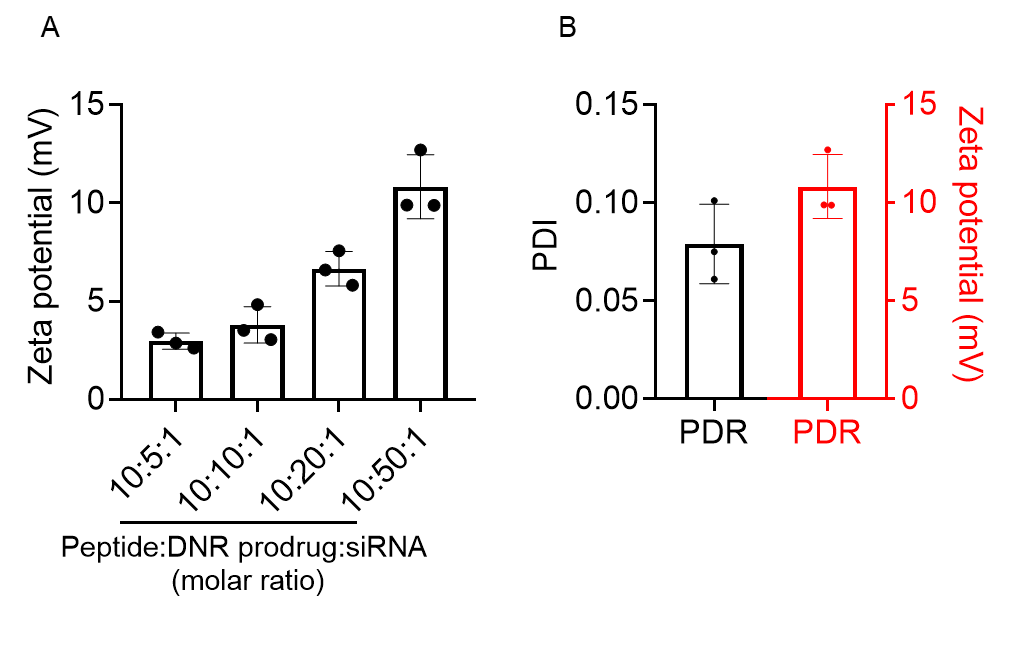


**Figure S4.** A) Zeta potential of PDR formulations with different component ratios, as determined by dynamic light scattering (DLS). B) Polydispersity index (PDI) and Zeta potential of the optimized PDR formulation (the molar ratio of Peptide:DNR-prodrug:siRNA=10:50:1), measured by DLS.


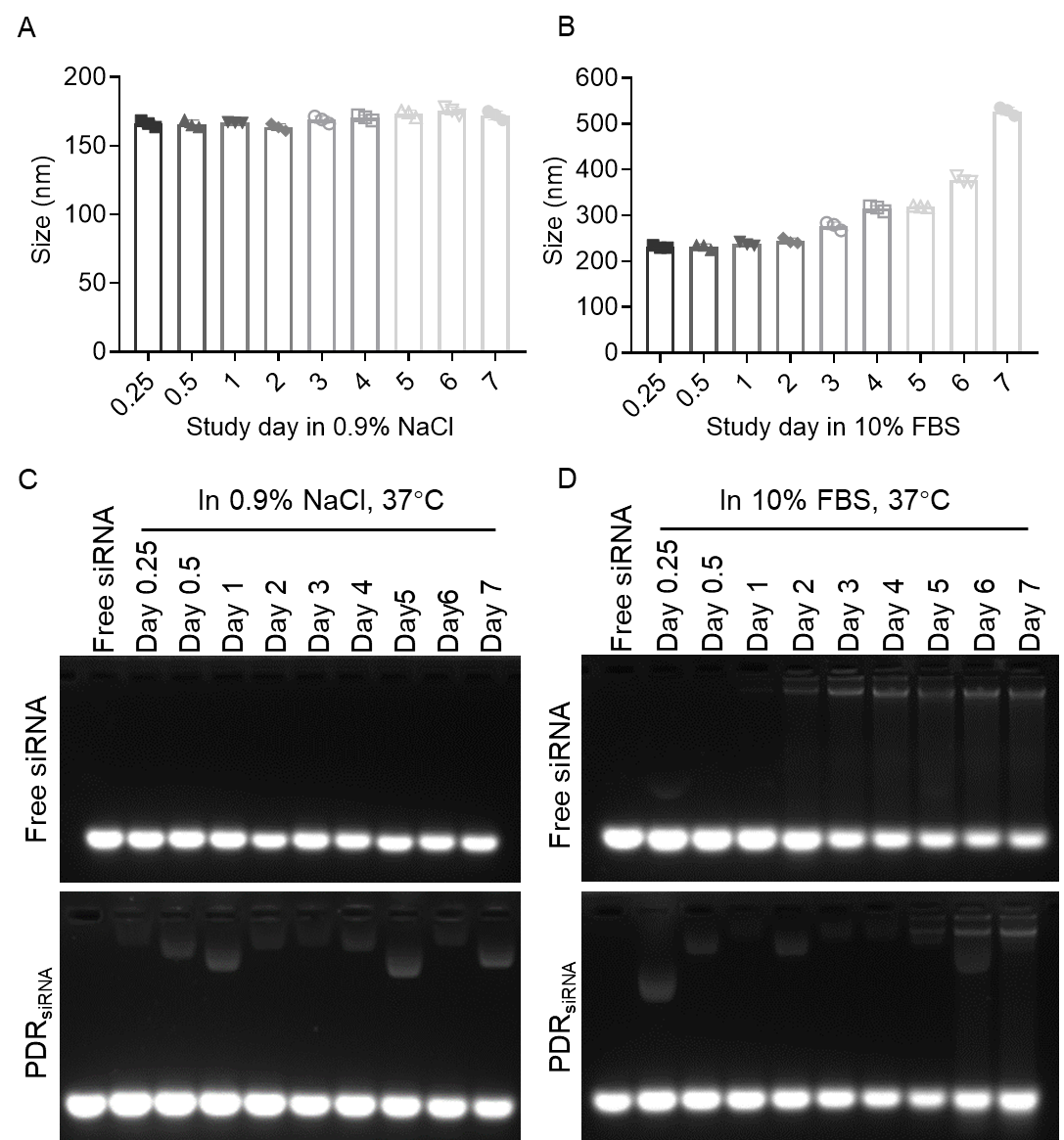


**Figure S5.** Evaluation of Physiological Stability of PDR Nanoparticles. A-B)The change in particle size of PDR nanoparticles incubated in saline (A) and 10% FBS (B) at 37 °C (*n*=3).


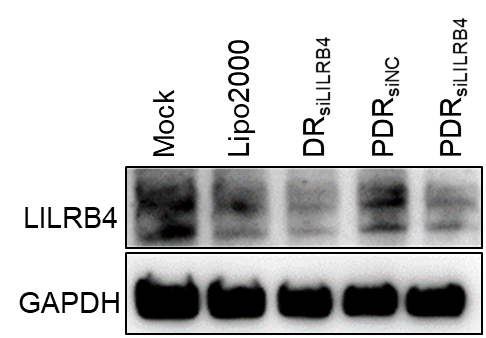


**Figure S6.** Western blot analysis of target protein expression in THP-1 cells transfected with Lipo2000_siLILRB4_, DR_siLILRB4_, PDR_siNC_, and PDR_siLILRB4_. siRNA was transfected at concentration of 100 nM.

**
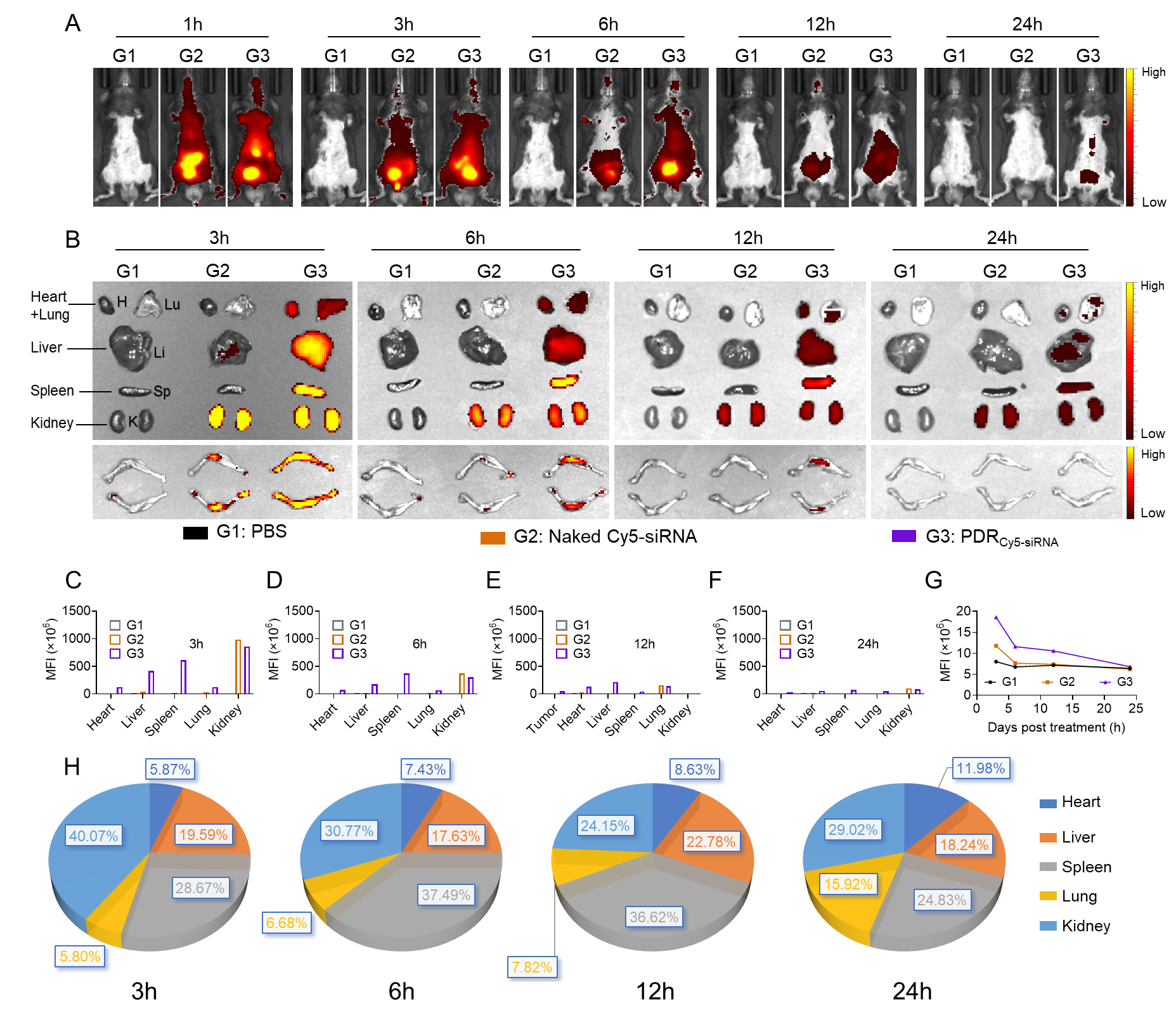
**

**Figure S7.** *In vivo* Biodistribution of PDR Nanoparticles. A) Whole-body fluorescence imaging of mice at various time points after intravenous injection of PDR nanoparticles. The injection dose of Cy5-siRNA was 1 mg/kg. B) Fluorescence distribution in major organs (heart, liver, spleen, lung, kidney, and bone) at specified time points post-injection. C-F) Quantitative analysis of fluorescence signals in different organs at indicated time points. G) Time-dependent curve of fluorescence intensity in the bone marrow of mice. H) Distribution of PDR nanoparticles in the heart, liver, spleen, lung, and kidney at corresponding time points.

**
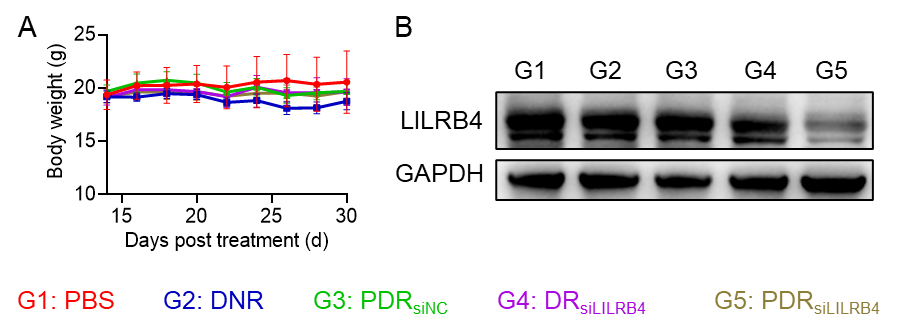
**

**Figure S8.** A) Body weight monitoring of mice during the treatment period. B) Expression of LILRB4 protein in mouse liver at the end of treatment.

**
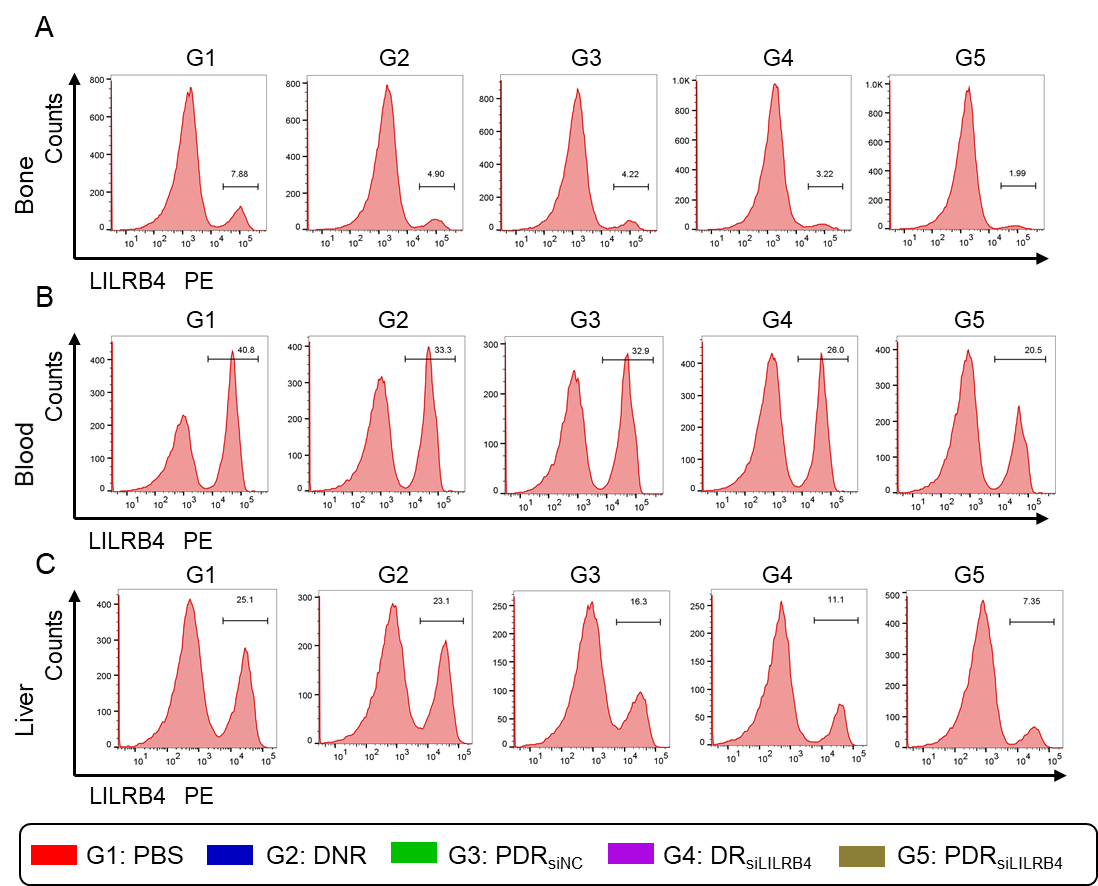
**

**Figure S9.** Assessment of leukemia infiltration efficiency *in vivo*. At the end of treatment, the infiltration of leukemia cells in the bone (A), blood (B), and liver (C) of mice receiving different treatments.


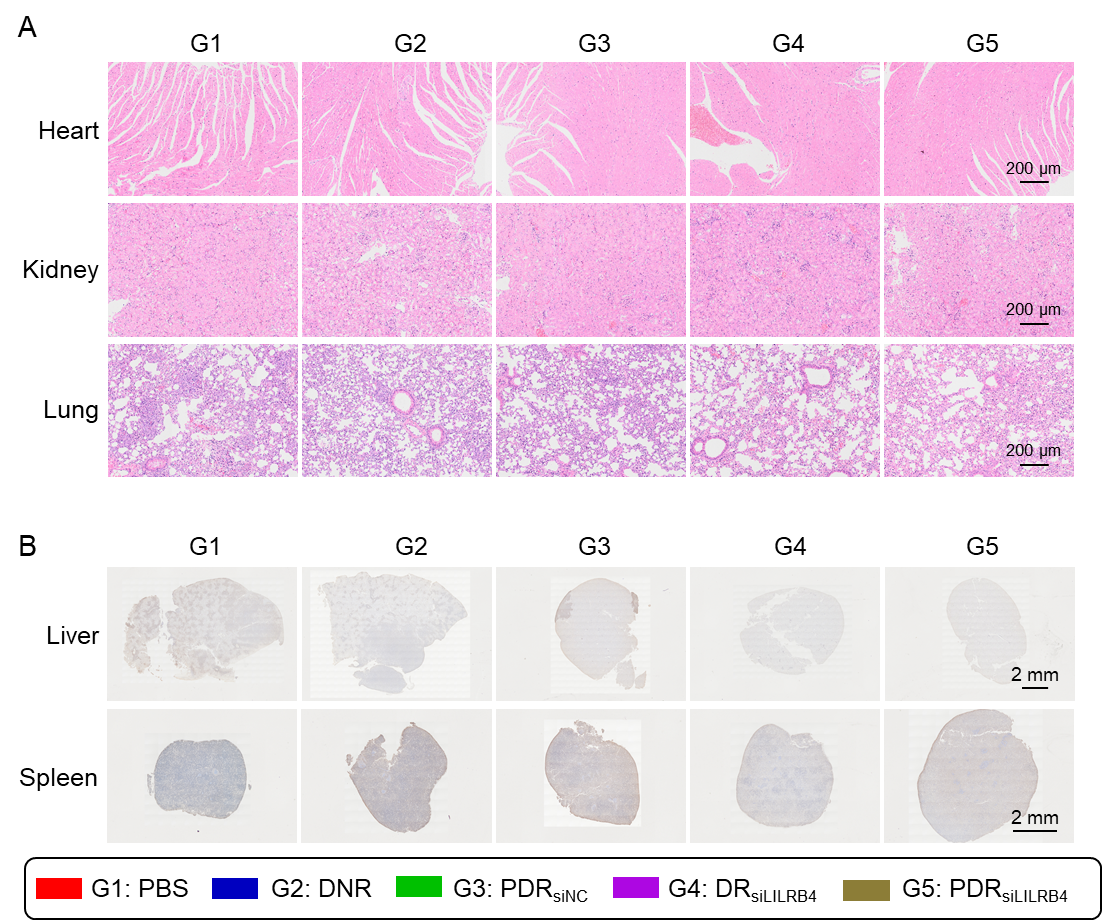


**Figure S10.** Assessment of leukemia infiltration efficiency *in vivo*. A) H&E staining of the heart, kidney and lung. Scale bars, 200 µm. B) Ki67 staining of the liver and spleen. Scale bars, 2 mm.
